# Cytokine Adsorption in Cardiac Surgery: where do we stand?

**DOI:** 10.21470/1678-9741-2019-0480

**Published:** 2020

**Authors:** Rohan Magoon, Manpal Loona, Jasvinder Kaur Kohli, Ramesh Kashav

**Affiliations:** 1 Department of Cardiac Anaesthesia, Atal Bihari Vajpayee Institute of Medical Sciences (ABVIMS) and Dr. Ram Manohar Lohia Hospital, Delhi, India.; 2 Department of Cardiothoracic Vascular Surgery, Atal Bihari Vajpayee Institute of Medical Sciences (ABVIMS) and Dr. Ram Manohar Lohia Hospital, Delhi, India.

Cardiopulmonary bypass (CPB) is inexorably linked to varying degrees of inflammatory response activation, mediated by diverse cytokines. A dysregulated cytokine inflammatory response, or a *cytokine storm*, results in significant systemic hypotension, culminating as multi-organ failure (MOF). Considering the association of the cytokine surge with adverse postoperative outcomes and heightened morbidity and mortality^[1]^, it has been proposed that the extracorporeal cytokine removal presents the potential of improving the outcomes across heterogeneous clinical scenarios characterized by lethal hyperinflammation.

In this context, various blood purification techniques (BPTs) have evolved for the maintenance of an immune homeostasis. These techniques include (but are not limited to): hemofiltration, continuous high-volume hemofiltration, hemoperfusion, plasmapheresis, high cutoff membranes, and adsorption. There is a considerable recent literature on the application of hemoadsorption (HA) therapy in acute inflammatory cytokine release syndrome attributable to sepsis, CPB, and/or autoimmune diseases^[2-5]^. HA therapy comprises the blood or plasma passage through a cartridge wherein the molecules are removed as a result of binding to the biocompatible sorbent. CytoSorb (CS) (CytoSorbents Corporation, New Jersey, United States of America) is a novel, single-use, polymer bead-based HA cartridge containing polyvinylpyrrolidone coated polystyrene divinylbenzene beads, capable of removing the mid-molecular weight solutes based on the combination of size exclusion and the underlying hydrophobic interactions. It has been approved in Europe since 2011 and can be utilized alone or in combination with renal replacement therapy (RRT), CPB machine, or extracorporeal membrane oxygenation (ECMO).

Albeit a sound theoretical premise, the initial literature on the application of HA-based cytokine removal in cardiac surgical cohort has yielded inconsistent results, in contrast to the more encouraging set of studies in septic patients. In a retrospective evaluation of a series of 16 patients with post-CPB systemic inflammatory response syndrome (SIRS) and acute kidney injury, Träger et al. reported a reduction in pro-inflammatory interleukin levels (interleukin-6,8) approximating the baseline levels, improved hemodynamic profile, and declined vasopressor dose requirements following the combination of CS therapy with the conventional RRT^[2]^. Similar findings were also outlined with the use of CS therapy in setting of infective endocarditis (IE)^[3]^. On the other hand, Bernardi et al. and Poli et al. failed to demonstrate a postoperative pro-inflammatory cytokine reduction or an altered perioperative course with CS therapy in their prospective studies incorporating cardiac surgical patients predisposed to medium and high risk of post-CPB complications, respectively^[6,7]^. However, Calabrò et al. depicted a reduction in 30-day mortality and vasopressor requirements with CS treatment in critically sick patients suffering from cardiogenic shock and MOF^[4]^. Nemeth et al. also reported reduced vasopressor dose and RRT requirement following HA in patients undergoing orthotopic heart transplantation (OHT)^[5]^.

A number of caveats on the application of CS in cardiac surgical arena surface on evaluating the available literature in close conjunction to the peculiarities of the CS therapy. Firstly, the inconsistency of postoperative cytokine decline in the aforementioned studies is explained by the concentrationdependent effects of CS treatment, wherein a marked cytokine decline with CS therapy manifests in settings of profound cytokinemia (critically sick patients, prolonged CPB, SIRS, IE, OHT, ECMO) compared to insignificant cytokine reduction in the healthier surgical subset accounting only for modest cytokine elevations. Thus, baseline cytokine levels, treatment duration, and frequency intricately impact the cytokine reduction extent. Secondly, the HA technique offers peculiar advantages over other BPTs in the form of lack of reliance on the removal of fluids for toxin-clearance, minimizing the fluid shifts attributable to conventional hemodiafiltration^[8]^. A considerably large surface area of the sorbent adds to the efficiency of the technique and the recent description of CS-based ticagrelor adsorption is particularly helpful in setting of an emergency cardiac surgery^[9]^. Despite the initial studies outlining the technical feasibility of incorporating CS cartridges in CPB machines and ECMO circuits, the maximal allowable flows through the cartridge and availability of standardized connections need to be considered for the safe integration of the cartridges in the complex circuit assembly. At the same time, the patients receiving HA therapy need to be closely monitored for alterations in antibiotic, platelet, and albumin levels. Moreover, the differential effect of HA treatment on the proinflammatory and the anti-inflammatory cytokines merit further evaluation. Lastly, the results of on-going large multi-centric trials on CS treatment in cardiac surgery are ardently awaited.

To conclude, cytokine adsorption opens a new promising frontier in the on-going endeavours aimed at combating post-CPB SIRS. However, an improved characterization of the patient cohort expected to benefit the most can add to the clinical meaningfulness and cost-effectiveness of the recent cytokine adsorption therapy.
